# A novel germline PAX5 single exon deletion in a pediatric patient with precursor B-cell leukemia

**DOI:** 10.1038/s41375-023-01991-0

**Published:** 2023-08-05

**Authors:** N. van Engelen, M. Roest, F. van Dijk, E. Sonneveld, R. Bladergroen, S. V. van Reijmersdal, V. H. J. van der Velden, P. G. Hoogeveen, W. A. Kors, E. Waanders, M. C. J. Jongmans, R. P. Kuiper

**Affiliations:** 1 Princess Máxima Center for Pediatric Oncology, Utrecht, The Netherlands; 2 Department of Genetics, Radboud University Medical Center, Nijmegen, The Netherlands; 3 Department of Immunology, Erasmus MC, University Medical Center Rotterdam, Rotterdam, The Netherlands; 4 Department of Genetics, Utrecht University Medical Center, Utrecht University, Utrecht, The Netherlands

**Keywords:** Cancer genetics, Haematological cancer

## To the Editor:

Germline investigation in rare families with multiple affected individuals and large cohorts of pediatric patients with acute lymphoblastic leukemia (ALL) has resulted in the discovery of a growing number of leukemia predisposing genes [1, 2]. A rare leukemia predisposition syndrome is caused by germline mutations in *PAX5*. Paired box 5 (PAX5) encodes for a paired box domain transcriptional factor essential for B-cell development [3]. Approximately 30% of the pediatric patients with B-cell precursor acute lymphoblastic leukemia (BCP-ALL) harbor a somatic heterozygous loss-of-function alteration in *PAX5* [4].

Three germline missense variants in *PAX5* have been described in seven families with a high incidence of BCP-ALL [5–9]. The first germline *PAX5* missense variant c.547G>A (p.Gly183Ser) was identified in four unrelated families [5, 6, 9]. Functional testing showed that the *PAX5* p.Gly183Ser variant has significantly reduced activity and results in deregulation of target genes, although the effect was milder than non-functional *PAX5* mutants [5]. A germline *PAX5* missense variant affecting the same hotspot, c.547G>C (p.Gly183Arg), has been reported in one family [9]. Two unrelated families were found to carry a germline c.113G>A (p.Arg38His) variant [7, 8]. Functional studies showed that *PAX5* p.Arg38His is also a hypomorphic variant resulting in incomplete B-cell differentiation and is sufficient to predispose to leukemia [8]. In all families, the susceptibility to BCP-ALL is inherited in an autosomal dominant pattern with incomplete penetrance. In the leukemia of the affected family members a second somatic alteration was detected in *PAX5*, either by loss of heterozygosity (LOH) or a second somatic mutation.

We report a novel germline *PAX5* alteration, a deletion including exon 6, in a boy who developed t(1;19)(q23;p13) (TCF3::PBX1) rearrangement-positive BCP-ALL at the age of 5. Somatic *PAX5* aberrations are detected in ~20% of the cases with TCF3::PBX1 BCP-ALL [10]. The patient was stratified in the standard risk group of the Dutch Childhood Oncology Group (DCOG) ALL11 treatment protocol and completed treatment without severe complications. He has been in follow up for three years. His father was diagnosed with acute undifferentiated leukemia at the age of 9 months. Cytogenetic testing of the leukemia of the father was not performed at time of diagnosis.

Because of the family history, the patient was referred to a clinical geneticist. He had no relevant medical history, showed normal growth and development, and had no skin abnormalities. Germline testing of the patient and his parents was performed using SNP-based copy number analysis (SNP-array) and whole exome sequencing (WES). This revealed a 14-kb loss of chromosome 9p13.2 encompassing exon 6 of *PAX5* in the patient, which was absent in both parents. Using multiplex-ligated probe amplification (MLPA), we detected this deletion in full-clonal heterozygous state in multiple DNA samples from the patient, including DNA form two blood samples, collected at different timepoints after complete remission, mesenchymal stem cells, cultured from a bone marrow sample at time of complete remission, and two independently harvested buccal swab samples. This confirms the germline status of the *PAX5* exon 6 deletion in the child (Supplementary Fig. 1).

**Fig. 1:**
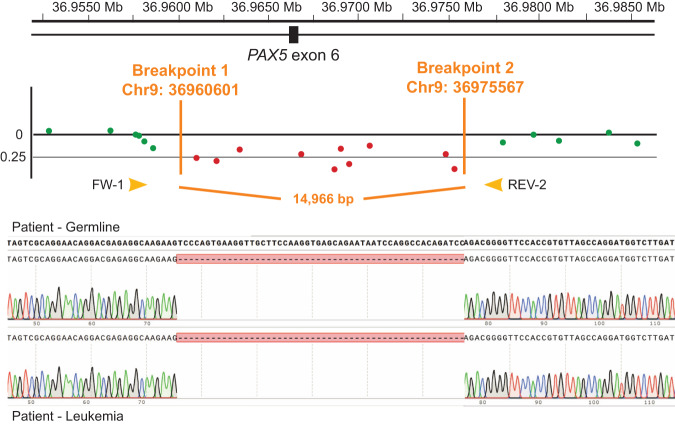
A germline *PAX5* deletion in a boy with leukemia. A schematic overview of the determination of the exact breakpoints of the deletion based on the results from the SNP-array. Sanger sequences of DNA derived from a remission and leukemia sample of the patient, showing the breakpoints of the *PAX5* exon 6 deletion in the sequence.

The apparent absence of this intragenic *PAX5* deletion in the father was unexpected, and we therefore tested whether he carried this deletion in a mosaic state. Unfortunately, a leukemia or historical remission sample from the father, which could have revealed presence of the *PAX5* deletion at time of diagnosis, was not available. Genotype analysis of SNPs directly flanking the breakpoints revealed that the *PAX5* deletion had occurred on the paternal allele in the child. Therefore, we developed a breakpoint-spanning PCR, to enable sensitive detection of the deletion in DNA of the father in (low) mosaic state. However, whereas this breakpoint-spanning fragment revealed the exact location and size of the *PAX5* deletion (Fig. 1), no deletion could be detected in blood and buccal cells of the father even with 60 ng of genomic DNA (~10,000 cells) as input, suggesting a *de novo PAX5* deletion event in the child. Of note, we also did not identify any aberrations in the father at or in 1000-bp surrounding regions of the *PAX5* deletion breakpoint positions which may have caused the deletion in the boy. Despite the high sensitivity of the breakpoint-spanning PCR, and absence of the deletion in both blood and buccal swap from the father, we cannot exclude that the germline *PAX5* deletion was present in the father below detection level and/or that a mosaic clone has been overgrown over time. To exclude the presence of an additional causative germline variant that was inherited from the father, we re-analyzed all sequence and copy number variants in the patient-parents’ WES trio datasets, but no plausible alternative candidates were identified.

The *PAX5* deletion causes a frameshift with premature stop in exon 7, resulting in a truncated protein with loss of the C-terminal transactivating and inhibitory domains. The deletion is not reported in the population databases for common structural variants DGV and dbVar [11]. *PAX5* has a pLI of 1.0 corresponding with an intolerance to loss-of-function variants. In RNA sequencing data from our patient and three patients with a somatic *PAX5* exon 6 deletion, we observed that the transcript lacking exon 6 is expressed and that the levels of expression of both alleles is similar. This suggests that nonsense mediated decay of the mutated transcript did not occur. Furthermore, Mullighan and coworkers earlier demonstrated the expression of truncated PAX5 proteins as a consequence of in-frame and out-of-frame partial gene deletions, suggesting a loss-of-function effect [4]. In previously described patients with germline *PAX5* missense variants, a second somatic hit in *PAX5* was observed [6, 8, 9]. We performed Sanger sequencing of the *PAX5* exons in a leukemia sample of the patient and performed SNP array-based copy number analysis on this sample, but did not observe a second alteration in *PAX5*. The only somatic aberration shared with the previously reported patients was a deletion in the *CDKN2A/B* locus, which was present in mosaic state in our patient (Supplementary Table 1).

**Fig. 2:**
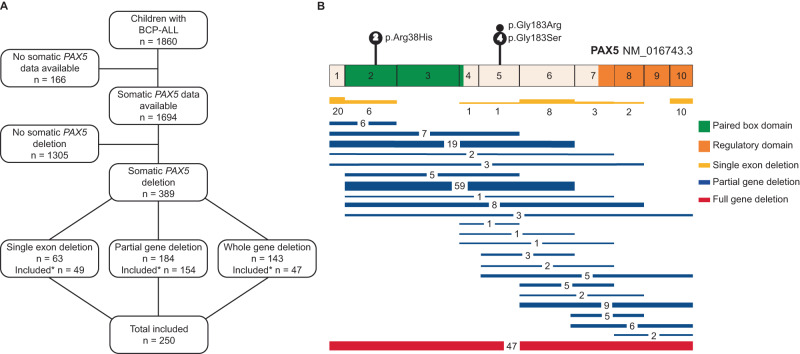
Information regarding the BCP-ALL cohort. **A** An overview of the cohort selection. *These patients were included in the analyses based on the somatic data and the availability of a remission sample. **B** A schematic overview of the PAX5 protein (NM_16743.3). The known germline *PAX5* variants are displayed on top of the protein overview. The somatic deletions in all included patients (*n* = 250) are displayed below the protein overview, with (a) the amount of somatic single exon deletions for each exon (yellow), (b) the amount of each somatic partial gene deletion (blue), and (c) the amount of somatic whole gene deletions (red).

In many centers, *PAX5* copy number aberrations in ALL are routinely tested by MLPA on DNA of leukemic samples only. For this reason, germline *PAX5* deletions could potentially be misinterpreted as somatic aberrations. We therefore selected BCP-ALL patients that were tested positive for *PAX5* deletions in the leukemia samples and screened for these deletions in remission samples of the same patients. An MLPA dataset of 1694 Dutch BCP-ALL patients revealed *PAX5* deletions in 389 (23%) of cases. We prioritized partial and intragenic *PAX5* deletions because larger germline deletions, encompassing multiple genes, are likely to present with more complex syndromic features. We identified 247 patients (14%) with an intragenic or partial *PAX5* deletion (Fig. 2A, B), which is in line with previous reports [4, 12]. For 44 patients no remission sample was available, resulting in a cohort of 203 remission samples that were tested for *PAX5* germline deletions by MLPA. In addition, from the 142 whole gene deletions identified in the cohort (8%), we included 47 available samples. In total, MLPA was performed on 250 remission samples. Three samples were excluded because of inconclusive results. No additional patients with a germline *PAX5* deletion were identified, indicating that this is a rare event.

BCP-ALLs resulting from germline missense *PAX5* aberrations mostly acquired a somatic *PAX5* deletion as a second hit. Therefore, we used the same cohort of remission samples from 250 *PAX5*-deleted cases to screen for germline point mutations using a smMIP-based panel encompassing all exons of *PAX5*. Three patients were identified with a variant of unknown significance (Supplementary Table 2), but no known or novel (likely) pathogenic germline *PAX5* variants were found.

Recently, germline *PAX5* alterations have been described in individuals with autism spectrum disorder [13]. Gofin et al. described sixteen individuals with a germline *PAX5* alteration: five individuals with a deletion including the *PAX5* gene, three individuals with a loss-of-function variant, and eight individuals with a missense variant. None of these patients, aged between 20 months and 24 years (median 10.5 years), developed leukemia. Our patient has not been diagnosed with autism spectrum disorder, nor was this reported in the families with a germline *PAX5* missense variant. The fact that none of the individuals with deleterious germline variants in *PAX5* in the study by Gofin et al. appears to have developed leukemia, may suggest an incomplete penetrance for cancer development as is also seen in the leukemia families with a germline *PAX5* missense variant. Larger cohort studies are required to investigate the penetrance for cancer in individuals with a germline *PAX5* alteration.

In conclusion, we present the first child with a BCP-ALL and a de novo germline *PAX5* exon 6 deletion leading to a truncated protein. Germline *PAX5* deletions have not yet been described in patients with BCP-ALL but considering the recurrence of somatic *PAX5* deletions in BCP-ALL, the germline deletion in our patient likely contributed to leukemia development.

## Supplementary information

Supplementary data

## Data Availability

The datasets for the cohort study generated during and/or analyzed during the current study are available from the corresponding author on reasonable request.
