# Supplementary material for: A novel germline PAX5 single exon deletion in a pediatric patient with precursor B-cell leukemia
Source: Leukemia. 2023 Aug 5;37(9):1908–11. doi: 10.1038/s41375-023-01991-0 (PMC10457179; doi:10.1038/s41375-023-01991-0)
Supplement: Supplementary file 1 — Supplementary data [file 41375_2023_1991_MOESM1_ESM.docx]

# Supplementary data

**Summary**

This supplementary data file contains:

1. The methods used to conduct the research described in the manuscript.
2. Supplementary Figure 1 with the MLPA results of the *PAX5* gene illustrating the loss of *PAX5* exon 6 in all germline samples from the patient from different origin.
3. Three supplementary tables:
   1. Supplementary Table 1: SNP-array results from the leukemia at diagnosis of the index patient.
   2. Supplementary Table 2: Variants of unknown significance detected by targeted sequencing.
   3. Supplementary Table 3: Primer sequences breakpoint spanning PCR.

**Methods**

Clinical ascertainment and consent

The index patient was identified in a diagnostic setting at the Princess Máxima Center for Pediatric Oncology in Utrecht, The Netherlands. The parents provided written informed consent for publication of their data and of their sons’ data.

For the cohort screening, we included pediatric ALL patients treated according to the Dutch Cancer Oncology Group (DCOG) protocols ALL8, ALL9, ALL10 or ALL11 in whom a somatic single exon or partial gene deletion in *PAX5* was confirmed. In addition, we selected a group of patients with a somatic *PAX5* whole gene deletion. From the included patients, pseudo-anonymized DNA samples at time of remission were collected. Samples were considered ‘remission samples’ if they had a Minimal Residual Disease (MRD) value of ≤10^-3^ or a blast percentage of < 1%, as defined by morphology and/or flowcytometric immunophenotyping. Samples were requested and collected from the DCOG biobank, since May 2018 known as the Princess Máxima Center for Pediatric Oncology biobank. In accordance with the Declaration of Helsinki, written informed consent was obtained from all patients and/or their legal guardians before enrollment in this biobank. The biobank review board approved the use of samples for this study (PMCLAB2020.132).

Single Nucleotide Polymorphism (SNP) Array

SNP array was performed on DNA isolated from leukemic bone marrow cells and a blood sample at time of remission from the patient and blood samples from the parents, using a CytoSNP-850K BeadChip SNP array (Illumina, San Diego, CA, USA). The data analysis was performed using NxClinical software (BioDiscovery, Los Angeles, CA, USA) on human genome build GRCh37/Hg19. CNV classification was performed using BENCH Lab CNV software (Agilent, Santa Clara, CA, USA).

Whole exome sequencing

The DNA samples were enriched with the Agilent SureselectxT Human All Exon 50Mb kit. Exome sequencing was performed on an Illumina Hiseq platform by BGI-Europe. Alignment was performed with BWA software package. The variant calling was performed with the GATK software for SNV and with the CoNIFER software for CNVs. The annotation was performed according to an inhouse annotation pipeline.

Polymerase Chain Reaction (PCR) & Sanger sequencing

Primers for the PCR were designed using Primer Express 3.0 and generated by Integrated DNA Technologies Europe (Leuven, Belgium). (Supplementary Table 3) To determine the breakpoints of the deletion we used the Phusion High-Fidelity DNA Polymerase Kit (ThermoFisher Scientific). To amplify the exons of *PAX5* and to amplify the regions surrounding the common SNPs we used the Taq DNA polymerase kit (Roche). The PCR product was run on an agarose gel. The PCR products were purified using the Exo-CIP^TM^ Rapid PCR Cleanup Kit (BioLabs) before they were sent to Macrogen Europe (Maastricht, The Netherlands) for Sanger sequencing. Sequence analysis was compared to the normal reference sequence (NM_016734.3) using Geneious Prime and SnapGene.

Determination of the parental allele

To determine the parental origin of the allele with the *PAX5* exon 6 deletion, we used informative common SNPs surrounding the deletion. Informative SNPs were defined as SNPs that are heterozygous in the patient and discordant homozygous in the parents. First, we identified informative SNPs outside of the breakpoints of the deletion in the patient and parents by PCR and Sanger sequencing. Second, we performed a PCR with a primer combination which would only amplify the allele with the deletion including the informative SNPs. The genotype of the SNPs on the amplified allele can be linked to one of the parents.

Multiplex-ligation dependent probe amplification (MPLA)

The detection of germline *PAX5* deletions was performed on bone marrow or peripheral blood samples taken at time of remission by Multiplex Ligation-dependent Probe Amplification (MLPA) using the SALSA MLPA P335 ALL-IKZF1-C1-1217 Probe Mix Kit (MRC Holland, Amsterdam, The Netherlands). The experiment was performed according to the manufacturers’ protocol with 70ng DNA. Fragment analysis was performed on an ABI-3730 analyzer (Applied Biosystems, Carlsbad, CA). The MLPA data was analyzed using Coffalyser® Software (MRC Holland, Amsterdam, The Netherlands). A rate below 0.75 was considered a mono-allelic deletion, a ratio between 0.75 and 1.3 was considered to represent a normal copy number, and a ratio higher than 1.3 was considered to represent a copy number gain.

Targeted Sequencing

For targeted sequencing we used a customized single molecule Molecular Inversion Probe (smMIP) panel. The probes were designed using the MIPgen pipeline at the Radboud University Medical Center Nijmegen, The Netherlands and covered all coding regions plus 20 nucleotides of the flanking introns [1]. The probes were manually checked in the UCSC browser and overlapping probes, probes with low quality scores or probes that were not unique were excluded. The selected probes were ordered at Biolegio BV (Nijmegen, The Netherlands). The smMIP protocol was performed as previously described [2]. Paired end sequencing (2x150bp) was performed on the Illumina MiniSeq platform using the MiniSeq System Mid-Output Kit (Illumina, San Diego, CA).

Reads were aligned to genome build GRCh37/Hg19 and analyzed using SeqNext software (v4.2.5, JSI, Ettenheim, Germany). Variant calling was performed based on single-molecule consensus reads [3]. Variants were annotated using VEP Ensemble Variant Effect Predictor. Variants were filtered on variant allele frequency ≥ 20% and GnomAD population frequency ≤ 1%. Remaining variants were individually analyzed based on pathogenicity scores and database classifications.

References

1. O’Roak BJ, Vives L, Fu W, Egertson JD, Stanaway IB, Phelps IG, et al. Multiplex targeted sequencing identifies recurrently mutated genes in autism spectrum disorders. Science. 2012; 338:1619–1622.

2. Hiatt JB, Pritchard CC, Salipante SJ, O’Roak BJ, Shendure J. Single molecule molecular inversion probes for targeted, high-accuracy detection of low-frequency variation. Genome Res. 2013; 23:843–854.

3. Yu J, Antić Ž, van Reijmersdal S V., Hoischen A, Sonneveld E, Waanders E, et al. Accurate detection of low-level mosaic mutations in pediatric acute lymphoblastic leukemia using single molecule tagging and deep-sequencing. Leuk Lymphoma. 2018; 59:1690–1699.


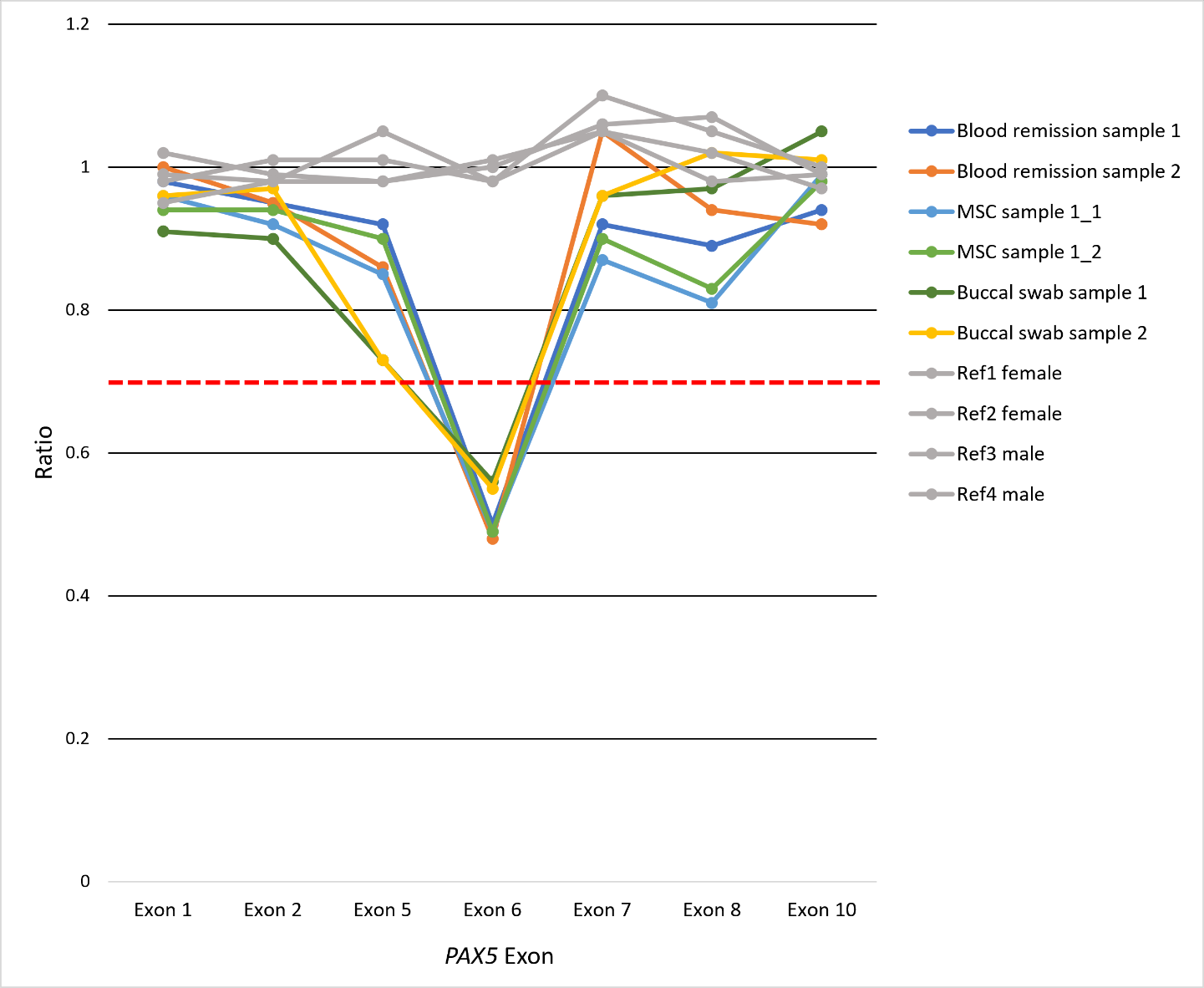


**Supplementary Figure 1:** The MLPA results of the *PAX5* gene illustrating the loss of *PAX5* exon 6 in all germline samples from different origin from the patient. Blood samples 1 and 2 were collected at day 79 (MRD negative) and day 182, respectively. MSC samples were cultured form a bone marrow sample from day 79 (MRD negative) and the isolated DNA was used in duplo for the MLPA. The buccal swab samples were collected after treatment when the patient was in complete remission. DNA from two individual samples was used for the MLPA.

**Supplementary Table 1:** SNP-array results from the leukemia at diagnosis of the index patient.

| **Chromosomal aberration** | **Variant allele frequency** | **Consequence** |
| --- | --- | --- |
| 1q23.1q23.3(158987941_164678924)x2 | 95% | aUPD |
| 1q23.3q44(164682390_249218992)x3 | 95% | t(1;19) *TCF3-PBX1* fusion |
| 5q21.1q21.3(101182591_107684259)x1 | 35% | *APC* loss |
| 5q22.1q22.3(109798056_113898201)x1 | 35% |  |
| 9p22.1p21.3(18929692_22364080)x1 | 20% | *CDKN2A/B* loss |
| 9p13.2(36960765_36975108)x1, | 50% | *PAX5* exon 6 deletion |
| 13q13.1q21.2(32651594_61905694)x1 | 35% | *RB1* loss |
| 19pterp13.3(260912_1615796)x1 | 90% | t(1;19) *TCF3-PBX1* fusion |

**Supplementary Table 2:** Variants of unknown significance detected by targeted sequencing.

| **Patient** | **Position (GRCh37)** | **cDNA** | **Amino acid change** | **Effect** | **GnomAD** | **CADD_Phred** | **SIFT** | **Polyphen** | **PhyloP100way** | **SpliceAI** | **LOVD** | **ClinVar** |
| --- | --- | --- | --- | --- | --- | --- | --- | --- | --- | --- | --- | --- |
| PAX5068 | Chr9:36840559A>T | c.1174T>A | p.(*392Argext*111) | Stoploss exon 10 /10 | - | 16.5 | - | - | 8.31 | Stoploss |  | NA |
| PAX5097 | Chr9:36882099C>T | c.914G>A | p.(Arg305His) | Missense exon 8/10 | 1.64x10-4 | 32 | Deleterious | Probably damaging | 7.41 | No effect | VUS | NA |
| PAX5189 | Chr9:36966554C>T | c.775G>A | p.(Glu259Lys) | Missense exon 6/10 | 6.57x10-6 | 22.6 | Tolerated | Probably damaging | 7.17 | No effect |  | NA |

**Supplementary Table 3:** Primer sequences breakpoint spanning PCR.

| **Primer name** | **Sequence** |
| --- | --- |
| PAX5_Breakpointspanning_FW1 | TGGGCGTCATACTCATCCATAAG |
| PAX5_Breakpointspanning_REV1 | GTACCCGGAATCCCATCTCTTAC |
| PAX5_Breakpointspanning_FW2 | GTGAGCAAGCAGAGGGACATG |
| PAX5_Breakpointspanning_REV2 | GTGTCAGGGGTTATGCTAAGT |
